# Incidence and risk factors for polypharmacy among elderly people assisted by primary health care in Brazil

**DOI:** 10.1186/s12877-023-04195-4

**Published:** 2023-08-04

**Authors:** Andréia Mascarelo, Ana Luisa Sant’Anna Alves, Siomara Regina Hahn, Marlene Doring, Marilene Rodrigues Portella

**Affiliations:** Postgraduate Program in Human Aging, University of Passo Fundo/UPF, BR 285 Km 292,7, Campus I, Bairro São José, Passo Fundo, RS CEP 99052-900 Brazil

**Keywords:** Aged, Polypharmacy, Primary health care

## Abstract

**Background:**

Polypharmacy is recognized as a global public health problem and one of the greatest challenges related to the aging population. Few studies have investigated the incidence and risk factors for polypharmacy among elderly individuals. These studies provided important information on the issue but were developed in high-income countries. This study investigates the incidence and risk factors for polypharmacy among elderly people assisted by primary health care over a period of 11 years.

**Methods:**

This was a census-based prospective longitudinal study that included people aged 60 years or older living in a small municipality in the state of Rio Grande do Sul, Brazil. The baseline occurred in 2010 and the second wave of the study occurred in 2021. The study population consisted of elderly individuals who did not use polypharmacy at baseline and were reinterviewed in 2021 (*N* = 128). Data collection in the first and second waves was performed through a household survey using a structured questionnaire. The dependent variable was polypharmacy, defined as the simultaneous use of 5 or more drugs. The independent variables included sociodemographic, health and functionality factors. For multivariate analyses, Poisson regression with robust variance was used, estimating the relative risk and 95% confidence intervals.

**Results:**

The incidence of polypharmacy was 46.1% in the 11-year period. The highest number of health problems was a risk factor for polypharmacy (RR = 1.177; 95% CI 1.093–1.267).

**Conclusions:**

The incidence of polypharmacy among elderly people assisted in primary health care in Brazil is high. The number of diseases is a risk factor for polypharmacy. These results have implications for future primary health care practices and may support the development of policies, actions and services aimed at reducing polypharmacy and promoting the rational use of drugs in the population at higher risk.

## Background

Polypharmacy is recognized as a global public health problem [1] and one of the greatest challenges related to the aging population, with a potential burden for elderly individuals, families and health systems [2].

With the increase in the number of elderly people and the greater availability of drugs observed worldwide, polypharmacy is becoming more frequent [3]. The prevalence of polypharmacy reported in the literature varies from 10% to approximately 90% in studies that considered different age groups, definitions of polypharmacy and geographic locations [2]. Similar to the situation worldwide, polypharmacy is prevalent in the Brazilian context [4–6], and evidence indicates an increasing trend in the use of polypharmacy among the elderly in more recent birth cohorts (1927–1937) when compared to those of previous cohorts (1916–1926) in Brazil [7].

Studies have associated the use of polypharmacy with negative health outcomes in elderly individuals, including adverse drug effects, harmful drug interactions, the use of potentially inappropriate medications, nonadherence to treatment, functional and cognitive declines, hospitalizations, high health costs and increased mortality [2].

However, few studies have investigated the incidence and risk factors for polypharmacy among elderly individuals [3, 8–11]. These studies provided important information on the issue but were developed in high-income countries and may not reflect the reality of the Brazilian context.

Knowing how polypharmacy develops over time and the factors that increase the risk of exposure is essential for the development and improvement of policies, actions and services aimed at the elderly age group. Therefore, the objective of this study was to investigate the incidence and risk factors for polypharmacy among elderly people over an 11-year period.

## Methods

A prospective, longitudinal, census-based study was conducted with people aged 60 years or older residing in the municipality of Coxilha in the state of Rio Grande do Sul, Brazil. The baseline occurred in 2010. Of the elderly recruited in 2010, those who did not use polypharmacy were included in this study and the elderly who died in the period, those who were not located and those who refused to participate in the continuity of the study in 2021 were excluded.

Data collection in the first and second waves was performed through a household survey, with face-to-face interviews, using exactly the same structured questionnaire both times [12]. The interviews in the first wave were conducted during the months of June and July 2010 by trained psychologists hired for the study. In the second wave, data collection occurred from August to December 2021 and was conducted by students of the Graduate Program in Human Aging of the University of Passo Fundo who underwent training. The interviewers’ training, in both moments, followed the same pattern and was conducted by the same team of researchers. The interviews were conducted individually, except when the presence of a substitute or auxiliary informant was necessary. Up to 3 attempts were made to visit the elderly participants on alternate days and times.

The dependent variable was polypharmacy, defined as the concomitant use of 5 or more drugs [1, 2]. The independent variables included sociodemographic information, health conditions, use of medications and functionality. All variables collected are detailed in Table 1.

**Table 1:** Study variables

**Variable**	**Categories or unit of measurement**	
**Sociodemographic**		
Gender	Male and female	
Age	In years	
Age group	60 to 69 years, 70 to 79 years, 80 to 89 years, 90 years or older	
Color	White and non-white	
Education	0–3 years and 4 or more years	
Area of residence	Urban and rural	
Resides	Alone and accompanied	
Marital status	With partner and without partner	
Retirement	Yes and No	
Income	0–2 minimum wages and 3 or more minimum wages	
**Health conditions**		
Health self-assessment	Positive and negative	
Health problems	Chronic pain	Yes and no
	Rheumatism	
	Asthma or bronchitis	
	Pulmonary emphysema	
	High blood pressure	
	Poor circulation	
	Diabetes *mellitus*	
	Obesity	
	Stroke or cerebral ischemia	
	Urinary incontinence	
	Constipation	
	Problem sleeping	
	Cataract	
	Back problems	
	Arthritis or arthrosis	
	Osteoporosis	
	Problems with nervousness	
	Heart problems	
	Anemia	
	Parkinson’s disease	
	Fecal incontinence	
	Cancer	
	Alzheimer’s disease	
	Depression	
Number of health problems	Number	
**Medications**		
Use of medications	Yes and No	
Number of medications	Number	
Pharmacological group	Group A - dietary tract and metabolism	Yes and no
	Group B - blood and hematopoietic organs	
	Group C - cardiovascular system	
	Group D - dermatological drugs	
	Group G - genitourinary system and sex hormones	
	Group H - systemic hormonal preparations, excluding sex hormones and insulins	
	Group J - anti-infective drugs for systemic use	
	Group L - antineoplastic and immunomodulatory agents	
	Group M - musculoskeletal system	
	Group N - nervous system	
	Group P - antiparasitic products, insecticides and repellents	
	Group R - respiratory system	
	Group S - sensory organs	
	Group V - several	
**Functionality**		
Cognitive state	With decline and without decline	
Basic activities of daily living	Independent and dependent	
Instrumental activities of daily living	Independent and dependent	

The age variable was categorized every 10 years because it is widely used in the scientific literature and subject to comparison.

Information on health problems was obtained through self-report, from a predetermined list [13].

The use of medications was verified through the following question: Do you use any medication? If so, what is the name? The names of the medications were obtained from the information provided by the elderly person or the substitute or auxiliary informant and, when possible, the prescription and/or packaging. From the name provided, the active substances of each drug that were listed were identified according to the first level (anatomical/pharmacological group) of the Anatomical Therapeutic Chemical – ATC [14].

To assess cognitive state, the Mini-Mental State Examination (MMSE) was used with the following cutoff points: 19 for illiterate, 23 for 1 to 3 years of schooling, 24 for 4 to 7 years of schooling and 28 for schooling over 7 years [15]. Based on the score obtained, the elderly participants were classified as having cognitive decline or no cognitive decline.

For basic activities of daily living (BADLs), the elderly participants were classified as independent or dependent using the Katz Index [16]. For instrumental activities of daily living (IADLs), the elderly participants were also classified as independent or dependent [17]. Those who were able to perform only one or more activities with assistance were classified as dependent.

The incidence of polypharmacy was calculated as the number of incident cases in the 11-year period among the unexposed elderly at the beginning of the study. The quantitative variables were described by measures of central tendency or position and variability, and normality was assessed using the Kolmogorov‒Smirnov test. To evaluate the association between polypharmacy and categorical variables, the Pearson chi-square test was applied. To compare the polypharmacy and nonpolypharmacy groups in relation to quantitative variables, the nonparametric Mann‒Whitney U test was used.

For multivariate analyses, Poisson regression with robust variance was used to calculate the relative risk and the respective 95% confidence intervals [18]. In the multiple model, the variables that had a *p* value ≤ 0.20 were considered in the bivariate analysis.

All analyses were performed using SPSS software (version 20) for Windows. The level of significance (⍺) adopted was 5%.

The study was approved by the Research Ethics Committee of the University of Passo Fundo, ruling number 2.189.982, in accordance with Resolution 466/2012 of the National Health Council. All study participants signed an informed consent form.

## Results

The population of this study consisted of 128 elderly people who did not use polypharmacy at baseline. Five (3.9%) elderly people needed help to answer the questionnaire. Among the participants, 59 (46.1%) transitioned to polypharmacy in the 11-year period. Variations in the incidence of polypharmacy were found between the different levels of education (0 to 3 years, 55.6% and 4 or more years, 41.8%), marital status (without a partner, 53.8% and with a partner, 40.8%), self-rated health (negative, 51.8% and positive, 41.2%) and age, as shown in Table 2.

**Table 2:** Sociodemographic, health and functional characteristics of elderly individuals (*N* = 128). Coxilha, RS, Brazil, 2010–2021

Variable	Without polypharmacy (*N* = 69)	Polypharmacy (*N* = 59)	N	*P*
	N (%) or median	N (%) or median		
Gender			128	0.457*
Female	31 (52.5)	28 (47.5)		
Male	38 (55.1)	31 (44.9)		
Age***	75.00 (73.00–79.00)	76.00 (74.00–81.00)	128	0.174**
Age group			128	0.508*
70 to 79 years	53 (57.0)	40 (43.0		
80 to 89 years	13 (44.8)	16 (55.2)		
90 years or older	3 (50.0)	3 (50.0)		
Color			128	0.548*
White	51 (53.7)	44 (46.3)		
Non-white	18 (54.5)	15 (45.5)		
Education			124	0.098*
From 0 to 3 years	20 (44.4)	25 (55.6)		
Four or more years	46 (58.2)	33 (41.8)		
Marital status			128	0.101*
Without partner	24 (46.2)	28 (53.8)		
With partner	45 (59.2)	31 (40.8)		
Income			124	0.326*
From 0 to 2 MW	49 (51.6)	46 (48.4)		
Three or more MW	17 (58.6)	12 (41.4)		
Area of residence			128	0.276*
Urban	41 (51.2)	39 (48.8)		
Rural	28 (58.3)	20 (41.7)		
Number of health problems***	3.00 (2.00–5.00)	6.00 (4.00–7.00)	128	< 0.001**
Health self-assessment			124	0.159*
Negative	27 (48.2)	29 (51.8)		
Positive	40 (58.8)	28 (41.2)		
BADLs			128	0.003*
Dependent	12 (33.3)	24 (66.7)		
Independent	57 (62.0)	35 (38.0)		
IADLs			128	0.002*
Dependent	32 (42.7)	43 (57.3)		
Independent	37 (69.8)	16 (30.2)		
Cognitive State			122	0.213*
With decline	26 (49.1)	27 (50.9)		
No decline	40 (58.0)	29 (42.0)		

*MW* Minimum wage, *BADLs* Basic activities of daily living, *IADLs* Instrumental activities of daily living

^*^Pearson’s chi-square test

^**^Mann‒Whitney U test

^***^Kolmogorov‒Smirnov test *p* < 0.05

In the bivariate analysis, there was an association between polypharmacy and the number of health problems, BADLs and IADLs (*p* < 0.05).

All elderly who made the transition to polypharmacy in the period had at least one chronic disease at the time of inclusion in the study, with a median of 4.00 health problems at baseline and 6.00 at follow-up. The median number of drugs consumed was 2.00 in 2010, rising to 6.00 in 2021 (*p* < 0.05).

**Fig. 1:**
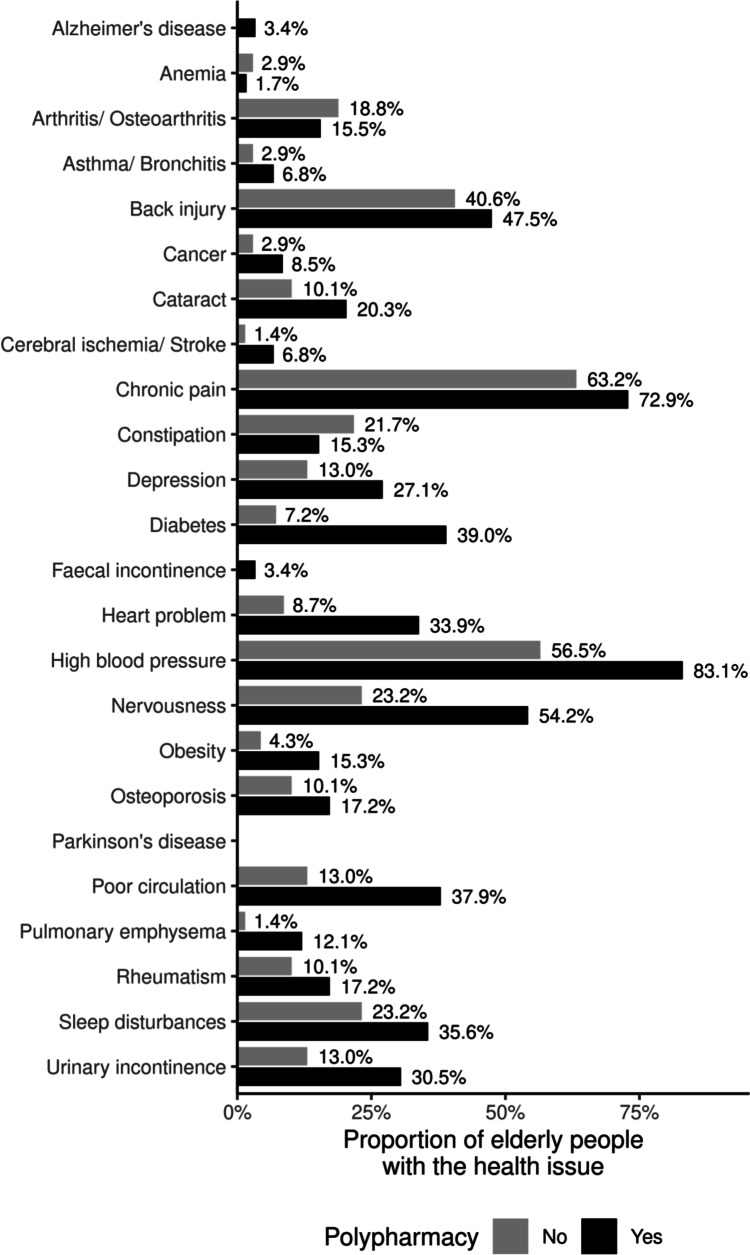
Chronic conditions among those exposed and not exposed to polypharmacy over the 11-year period

Figure 1 shows the occurrence of chronic conditions among those exposed and not exposed to polypharmacy over the 11-year period.

Polypharmacy was more frequent among the elderly who had high blood pressure (83.1%), chronic pain (72.9%), nervousness problems (54.2%), spine problems (47.5%) and diabetes mellitus (39.0%).

**Fig. 2:**
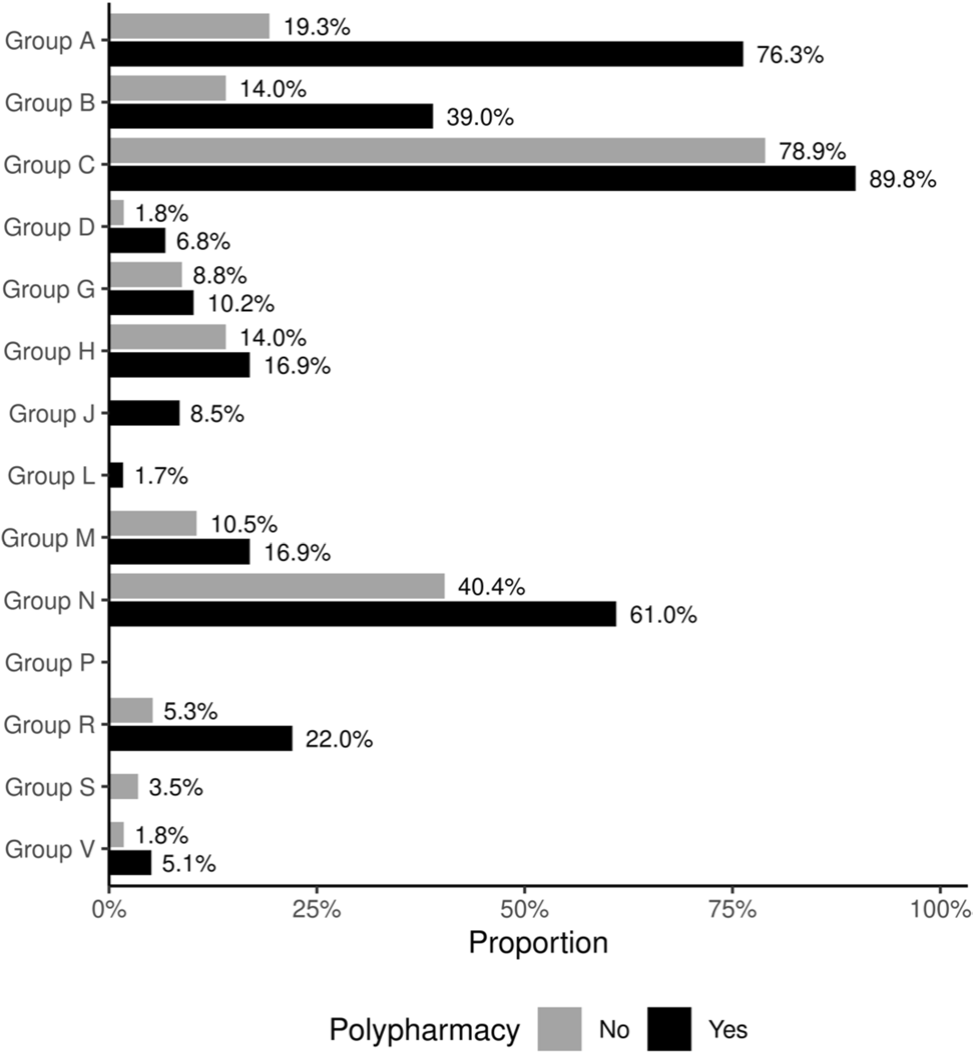
Medication use among those exposed and not exposed to polypharmacy in an 11-year period

Figure 2 shows the frequency of use of drug groups among elderly people exposed and not exposed to polypharmacy over the 11-year period. The main groups of drugs involved in incident polypharmacy were Group A, with action on the food tract and metabolism (76.3%), Group C, with action on the cardiovascular system (89.8%), and Group N, with action on the nervous system (61.0%). In particular, Group A showed the highest growth, in terms of relative numbers, in the group exposed to polypharmacy (76.3%) compared with the group not exposed to polypharmacy (19.3%), as shown in Fig. 2.

In the crude analysis, a significantly higher incidence of polypharmacy was observed among elderly people who had a higher number of health problems (*p* < 0.001) and who had some degree of dependence on BADL (*p* = 0.003) and IADL (*p* = 0.002) (Table 3).

**Table 3:** Incidence of and risk factors for polypharmacy among elderly individuals. Coxilha, RS, Brazil, 2010–2021

Variable	N (%)	*P*	RR^a^ (IC 95%)	RR^b^ (IC 95%)
Age	76.00	0.174*	1.014 (0.984–1.045)	1.020 (0.986–1.055)
Education		0.098**		
From 0 to 3 years	25 (55.6)		1.330 (0.920–1.923)	1.294 (0.913–1.833)
Four or more years	33 (41.8)		1.00	1.00
Marital status		0.101**		
Without partner	28 (53.8)		1.320 (0.912–1.911)	1.005 (0.674–1.498)
With partner	31 (40.8)		1.00	1.00
Number of health problems	6.00	< 0.001*	1.169 (1.104–1.237)	1.177 (1.093–1.267)
Health self -assessment		0.159**		
Negative	29 (51.8)		1.258 (0.860–1.839)	0.794 (0.547–1.152)
Positive	28 (41.2)		1.00	1.00
BADL		0.003**		
Dependent	24 (66.7)		1.752 (1.237–2.483)	1.100 (0.746–1.622)
Independent	35 (38.0)		1.00	1.00
IADL		0.002**		
Dependent	43 (57.3)		1.899 (1.207–2.989)	1.143 (0.685–1.907)
Independent	16 (30.2)		1.00	1.00

*CI* Confidence interval

^*^Mann‒Whitney U test

^**^Pearson’s chi-square test

^a^RR: Gross relative risk, Poisson regression with robust variance

^b^RR: Adjusted relative risk, Poisson regression with robust variance

In the adjusted model, only the number of health problems was associated with an increase in the incidence of polypharmacy (RR = 1.177; 95% CI 1.093–1.267). With each new health problem, there was an average increase of 17% in the incidence of polypharmacy (Table 3).

## Discussion

This study revealed that during an 11-year follow-up, polypharmacy affected 46.1% of elderly individuals.

Few studies have investigated the incidence of polypharmacy, both among the elderly and among the general population [3, 8–11]. A study that used data from 1.3 million elderly people aged 65 years or older in Denmark found an incidence of polypharmacy of 46.9% in 5 years of follow-up [10]. In Sweden, a study that included data from 1.7 million elderly individuals (age ≥ 65 years) found a polypharmacy incidence of 20% over a 3-year period [3]. In the Netherlands, a study that included data from 1544 elderly individuals from 1994 to 1997 revealed that almost 20% of the elderly developed polypharmacy (2 or more drugs) in the 4-year period [11]. Among Italian elderly (age ≥ 65 years), polypharmacy affected 1.4% of the elderly population in 2001, increasing to 2.8% in 2009 [9]. Another study that included data from the pharmacoepidemiological registry of Odense, Denmark, identified an incidence of 0.2% per month [8].

Our results, although obtained in a middle-income country, follow the trend observed in high-income countries, with a considerable level of incident polypharmacy among elderly people over time. However, it is prudent to consider that the different methodologies used in the studies, in addition to influencing the incidence of polypharmacy, make comparisons difficult. Studies on the subject in other countries involved different follow-up periods, age groups and definitions of polypharmacy. They mostly included large populations and used administrative data from dispensation and health insurance records. In this census study, we used data obtained during face-to-face interviews with elderly people living in a small municipality in southern Brazil and considered the drugs actually consumed. We found that almost a quarter of the elderly were exposed to polypharmacy at the time of inclusion [12] and that another 46.1% made the transition to polypharmacy over a period of 11 years, which suggests that most elderly individuals may be exposed to polypharmacy at some point in their life.

The high incidence of polypharmacy observed in this study may reflect both negative and positive aspects. On the one hand, it raises concern because polypharmacy has been associated with adverse health outcomes in elderly individuals, such as adverse drug reactions, frailty, physical and cognitive disabilities, hospitalizations and mortality [2, 19]. However, reviews have highlighted limitations in the articles published on the subject, such as problems related to confusion by indication, in which there were difficulties in separating the effects of polypharmacy from the underlying health problem for which the drugs were indicated [19]. Notably, most outcomes associated with polypharmacy are associated with preexisting multimorbidity [2].

This high incidence of polypharmacy, in the context studied, may indicate positive aspects. Because it is a low-income country with large social and economic disparities, the high incidence of polypharmacy may reflect general improvements in the Brazilian health system, which may have contributed to greater access of the population to health services and therefore to the means for diagnosis and treatment. In this study, all elderly people exposed to polypharmacy had at least one chronic disease at baseline. However, we observed an increase between 2010 and 2021 in the number of self-reported diseases (4.00 and 6.00) respectively, and in the number of medications consumed (2.00 and 6.00) respectively. Considering that they are self-reported diseases, it is inferred that they depend on the use of health services for the knowledge of their existence.

In Brazil, the National Health Survey showed that between 2013 and 2019, there was an increase in access by the Brazilian population, especially among elderly individuals, to health services and pharmaceutical care [20]. In fact, evidence points to improvements in the organization of health systems and technological advances as factors that contribute to the increase in drug prescription and polypharmacy rates because they provide early diagnosis of and treatment for chronic diseases [2, 21–23] and the discovery and development of new drugs [24].

The expansion of access to free pharmaceutical care in the country, made possible by the implementation and qualification of public health policies in recent years, seems to have a central role in the expansion of the use of medicines by the population. The National List of Essential Medicines (RENAME) is revised and expanded every 2 years and includes a comprehensive list of drugs indicated for the treatment of diseases prevalent in the country. These drugs must be funded by municipalities, states and the federal government and are offered free of charge to the population [25], a factor that may have contributed to the greater access to pharmaceutical assistance care and use of medications among the elderly in this study because people with free drug coverage have a higher risk of polypharmacy than do those who need to pay for pharmaceutical care [26]. However, evidence indicates inequalities in the access of the Brazilian population to health services and pharmaceutical care in different regions of the country [20] and discrepant results in the proportion of elderly individuals using polypharmacy [27]. In Brazil, although the health system is unique, one of its principles is political-administrative decentralization, with a single direction in each sphere of government and with emphasis on the decentralization of services to the municipalities, which are responsible, among other responsibilities, for the necessary financial support, according to the local epidemiological reality and the management of pharmaceutical assistance, including the purchase and dispensing of medicines for the population [25]. Differences in access to health services and pharmaceutical care in different regions of the country raise concern, as in places with elderly populations similar to the one in this study, issues related to public health management can compromise access to health services and medicines, reducing the possibilities of diagnosis and access to adequate treatment.

Recent studies have identified a higher risk of the development of polypharmacy among older adults [3, 10] and among divorced and widowed individuals than among married individuals [10]. In this study, we observed a higher incidence of polypharmacy among the elderly participants in the age group of 80 to 89 years but a reduction in the age group of 90 years or more. We also found that polypharmacy was more frequent among elderly individuals who did not have a spouse. However, we did not find significant differences in the incidence of polypharmacy between different age groups and marital status.

The main groups of drugs involved in incident polypharmacy were Group A, with action on the food tract and metabolism; Group C, with action on the cardiovascular system; and Group N, with action on the nervous system. This pattern is similar to that observed among elderly people using polypharmacy in other regions of Brazil [4, 5, 7] and in other countries [10, 21, 28] and may reflect the need for drug treatment of the most frequent health problems in groups exposed to polypharmacy: high blood pressure, chronic pain, nervousness problems, spine problems and diabetes mellitus. However, notably, the significant use of Group A drugs does not seem to be explained only by the occurrence of diabetes mellitus. Although in this study we did not investigate the substances consumed by elderly individuals, the results obtained and the available evidence suggest that the expressive use of Group A drugs by those exposed to polypharmacy could be related to the use of gastric protectors. Gastric protectors are commonly prescribed to elderly people who use many medications for preventive purposes [4], an indication that is not always made safe. These drugs are widely prescribed in primary health care in Brazil, especially for elderly individuals, without a clear indication and for prolonged periods of time, increasing the chances of exposure to the associated adverse effects [29] such as an increased risk of osteoporosis, Clostridium difficile colitis, interstitial nephritis and pneumonia [30].

In this study, the highest number of health problems was a risk factor for polypharmacy. Our findings are consistent with the literature that relates the highest number of chronic diseases to more frequent exposure to polypharmacy [3, 10]. This result is justified because during the 11 years of follow-up, the participants were aging and, therefore, more prone to the development of new chronic conditions, which requires, in most cases, drug therapy. People with coexisting chronic conditions usually have symptoms and deficiencies that require a more frequent interaction with health services [31], and in this scenario, drugs are the most common therapeutic intervention [1]. Thus, the need to manage multiple chronic conditions usually leads to higher drug consumption and polypharmacy [19]. In addition, patients with multiple diseases are usually under the care of different specialists, who naturally prioritize the approach of problems within their area of expertise, which may result in different prescriptions [24].

However, there are other underlying mechanisms that may influence the development of polypharmacy among elderly people with coexisting diseases. One of these mechanisms may have its origin in the national health policy, which profoundly influenced the management of chronic diseases in the country. As a strategy for the care of people with chronic disease, the Ministry of Health recommends to primary health care teams the use of clinical protocols and therapeutic guidelines, emphasizing that the use of these devices is related to better quality of care and more appropriate treatments and better health outcomes [32]. Along these lines, the Brazilian RENAME, with the aim of leveling the supply of care in the SUS, avoiding conflicts of conduct and promoting the rational use of drugs, was structured in accordance with the clinical protocols and therapeutic guidelines in force in the country [25]. However, these guidelines are usually directed to single diseases and rarely take multimorbidity into account, which is why elderly people with coexisting diseases may be prescribed various medications [23]. In addition, most studies on which the guidelines are based commonly exclude elderly people with multimorbidity, making their application potentially dangerous in elderly individuals with multiple chronic conditions [31]. Thus, the use of therapeutic guidelines may contribute to the development of polypharmacy among elderly people assisted by primary health care, as it is in the primary care environment, understood as the preferential contact of the elderly population with the health system, that most prescriptions for patients with long-term conditions occur [23].

Another mechanism is related to the different attitudes of prescribers regarding the challenge of treating elderly people with complex health problems. Some prescribers are more inclined to adopt clinical protocols as a basis for therapeutic management, while others consider the characteristics, priorities and preferences of the elderly for the recommendation of treatment [23]. Thus, the different behaviors of prescribers may result in different amounts of prescribed drugs. Nevertheless, in this context, factors such as the practice of defensive prescription, motivated by the concern with medico-legal issues and the pressure exerted by the patient for the prescription, may result in the indication of potentially unnecessary drugs [24], contributing to the use of more drugs and polypharmacy.

As strengths, we highlight that this study included all elderly individuals aged 60 years or older registered as residents in the municipality of Coxilha, RS, Brazil, in 2010 and revisited them in 2021, which limits the risk of selection bias. Based on the information obtained through face-to-face interviews, it was possible to investigate the use of medications effectively consumed by elderly individuals because studies that use data from administrative records may not reflect the actual use of medications. The performance of a longitudinal study revealed the incidence of polypharmacy over a period of 11 years, which makes this study one of the few to investigate the incidence of polypharmacy. The census character and long-term follow-up are the main strengths of this study.

Regarding the limitations, we did not evaluate the adequacy of the polypharmacy used by the elderly participants at the individual level, as this would require the clinical judgment of each case, which is difficult to do in studies involving many participants. We also did not evaluate the classes of drugs used by the elderly exposed to polypharmacy. Future research may contribute to the knowledge in the field by studying the classes of drugs responsible for incident polypharmacy as well as describing the adequacy of polypharmacy, so that we can obtain a deeper understanding of the subject.

## Conclusions

The incidence of polypharmacy among elderly people assisted in primary health care in Brazil is high. The number of diseases is a risk factor for polypharmacy. Our findings broaden understanding of the epidemiology of polypharmacy and have implications for future practice in primary health care, as they provide subsidies for the development of policies, actions and services aimed at promoting the rational use of medicines in populations at higher risk.

## Data Availability

Datasets used and analyzed during the current study are available from the corresponding author upon reasonable request.

## Abbreviations

BADLs: Basic activities of daily living
IADLs: Instrumental activities of daily living
RS: Rio Grande do Sul
SUS: Sistema Único De Saúde (Unified Health System)
RENAME: Relação Nacional de Medicamentos Essenciais (National List of Essential Medicines)
